# Supplementary material for: Therapeutic failure of multidrug therapy for leprosy: A retrospective case series in a hyperendemic Brazilian City
Source: PLoS Negl Trop Dis. 2025 Nov 25;19(11):e0013616. doi: 10.1371/journal.pntd.0013616 (PMC12646476; doi:10.1371/journal.pntd.0013616)
Supplement: S1 STROBE Checklist — https://www.strobe-statement.org/. (DOCX) [file pntd.0013616.s001.docx]

# S1 Checklist – STROBE Statement

This checklist ensures transparency and completeness in reporting observational studies, adapted to this retrospective case series of multibacillary leprosy patients.

## Title and abstract

Indicated the study design as a retrospective case series; provided an informative abstract with design, participants, and main findings.

## Background/rationale

Explained the scientific background and rationale for investigating persistent disease after multidrug therapy (MDT).

## Objectives

Stated specific objectives to describe clinical, histopathological, molecular, and inoculation findings in therapeutic failure cases.

## Study design

Described as a retrospective observational case series (complete cohort, no sample size calculation).

## Setting

Specified the reference center in Brazil, study period, and inclusion criteria.

## Participants

Reported eligibility criteria and numbers at each stage (n=131 included).

## Variables

Defined outcomes and key variables, including histopathology, qPCR, slit-skin smear BI/MI, and nude-mouse inoculation.

## Data sources/measurement

Detailed specimen collection, histological review, molecular assays, and inoculation protocol.

## Bias

Addressed potential biases related to retrospective design and missing data.

## Study size

Explained that all eligible patients were included; no formal sample-size calculation was performed.

## Quantitative variables

Described how laboratory variables were categorized and interpreted (e.g., BI, MI, CT < 40 threshold for qPCR).

## Statistical methods

Explained descriptive approach; proportions reported with exact binomial 95% confidence intervals.

## Participants (Results)

Reported numbers for each diagnostic modality with denominators reflecting available cases.

## Descriptive data

Summarized demographic and clinical characteristics in Table 1.

## Outcome data

Provided results for histopathology, qPCR, inoculation, and BI/MI at post-MDT index evaluation.

## Main results

Reported concordance across methods and described frequencies with confidence intervals.

## Other analyses

Explored agreement between histology and inoculation (Table 5).

## Key results

Summarized persistence of bacilli despite MDT and the dissociation between skin and nerve outcomes.

## Limitations

Acknowledged retrospective design, missing laboratory data, and absence of anti-PGL-1 serology.

## Interpretation

Placed findings in context of treatment monitoring and resistance detection.

## Generalizability

Discussed applicability to referral centers and implications for programmatic surveillance.

Attribution / License Information
This checklist was adapted from the STROBE Statement (Strengthening the Reporting of Observational Studies in Epidemiology). Source: https://www.strobe-statement.org
Licensed under a Creative Commons Attribution 4.0 International License (CC BY 4.0): https://creativecommons.org/licenses/by/4.0/
