# Supplementary material for: Therapeutic failure of multidrug therapy for leprosy: A retrospective case series in a hyperendemic Brazilian City
Source: PLoS Negl Trop Dis. 2025 Nov 25;19(11):e0013616. doi: 10.1371/journal.pntd.0013616 (PMC12646476; doi:10.1371/journal.pntd.0013616)
Supplement: S1 CARE Checklist — (DOCX) [file pntd.0013616.s002.docx]

# S2 Checklist – CARE Statement

This checklist ensures adequate reporting of clinical case series, adapted to the study on therapeutic failure in multibacillary leprosy.

## Title

Identified as a retrospective case series of therapeutic failure in multibacillary leprosy.

## Keywords

Included relevant terms: leprosy, multidrug therapy, therapeutic failure, qPCR, histopathology, nude-mouse inoculation.

## Abstract

Structured with background, methods, results, and conclusions.

## Introduction

Presented rationale and knowledge gap on persistent disease post-MDT.

## Patient information

Summarized demographics, clinical manifestations, and prior MDT exposure.

## Clinical findings

Described dermatological and neurological status at post-MDT evaluation.

## Timeline

Provided a visual figure of patient course and evaluations (S3 Figure).

## Diagnostic assessment

Detailed slit-skin smears, histopathology, molecular assays, and inoculation protocols.

## Therapeutic interventions

Not applicable; no new treatment regimen tested.

## Follow-up and outcomes

Reported persistent disease markers despite MDT, with dissociation between skin and nerve outcomes.

## Discussion

Addressed strengths, limitations, and implications for clinical management and surveillance.

## Patient perspective

Not applicable; retrospective design without direct patient interviews.

## Informed consent

Ethics approvals stated; waiver obtained for retrospective use of clinical samples.

Attribution / License Information
This checklist was adapted from the CARE Statement (Case Report guidelines). Source: https://www.care-statement.org
Licensed under a Creative Commons Attribution 4.0 International License (CC BY 4.0): https://creativecommons.org/licenses/by/4.0/
