# Supplementary material for: Therapeutic failure of multidrug therapy for leprosy: A retrospective case series in a hyperendemic Brazilian City
Source: PLoS Negl Trop Dis. 2025 Nov 25;19(11):e0013616. doi: 10.1371/journal.pntd.0013616 (PMC12646476; doi:10.1371/journal.pntd.0013616)
Supplement: S1 File — (DOCX) [file pntd.0013616.s003.docx]

**Patient treated with 24 doses of WHO-Regular MDT, without therapeutic response.**

One of the patients in this study was treated for 24 months with regular WHO-MDT. There was deterioration of neural function, and the nude mouse inoculation was positive after treatment, with biopsy material from the right common peroneal nerve. (a): Presence of anesthetic lesions on the back at the time of diagnosis, of the polymorphous erythema-like type. (b): Remission of lesions after 24 months of treatment with WHO-MDT. (c) and (d): Deterioration of neural function, with both soles of the feet completely anesthetized after treatment. Initial esthesiometry revealed only loss of sensitivity to the 2.0 g Semmes-Weinstein monofilament (purple).

Image credit**:** Andrea Maia Fernandes de Araújo Fonseca, adapted and licensed under [CC BY 4.0](https://creativecommons.org/licenses/by/4.0/?utm_source=chatgpt.com)
