# Supplementary figures and images for: Therapeutic failure of multidrug therapy for leprosy: A retrospective case series in a hyperendemic Brazilian City
Source: PLoS Negl Trop Dis. 2025 Nov 25;19(11):e0013616. doi: 10.1371/journal.pntd.0013616 (PMC12646476; doi:10.1371/journal.pntd.0013616)

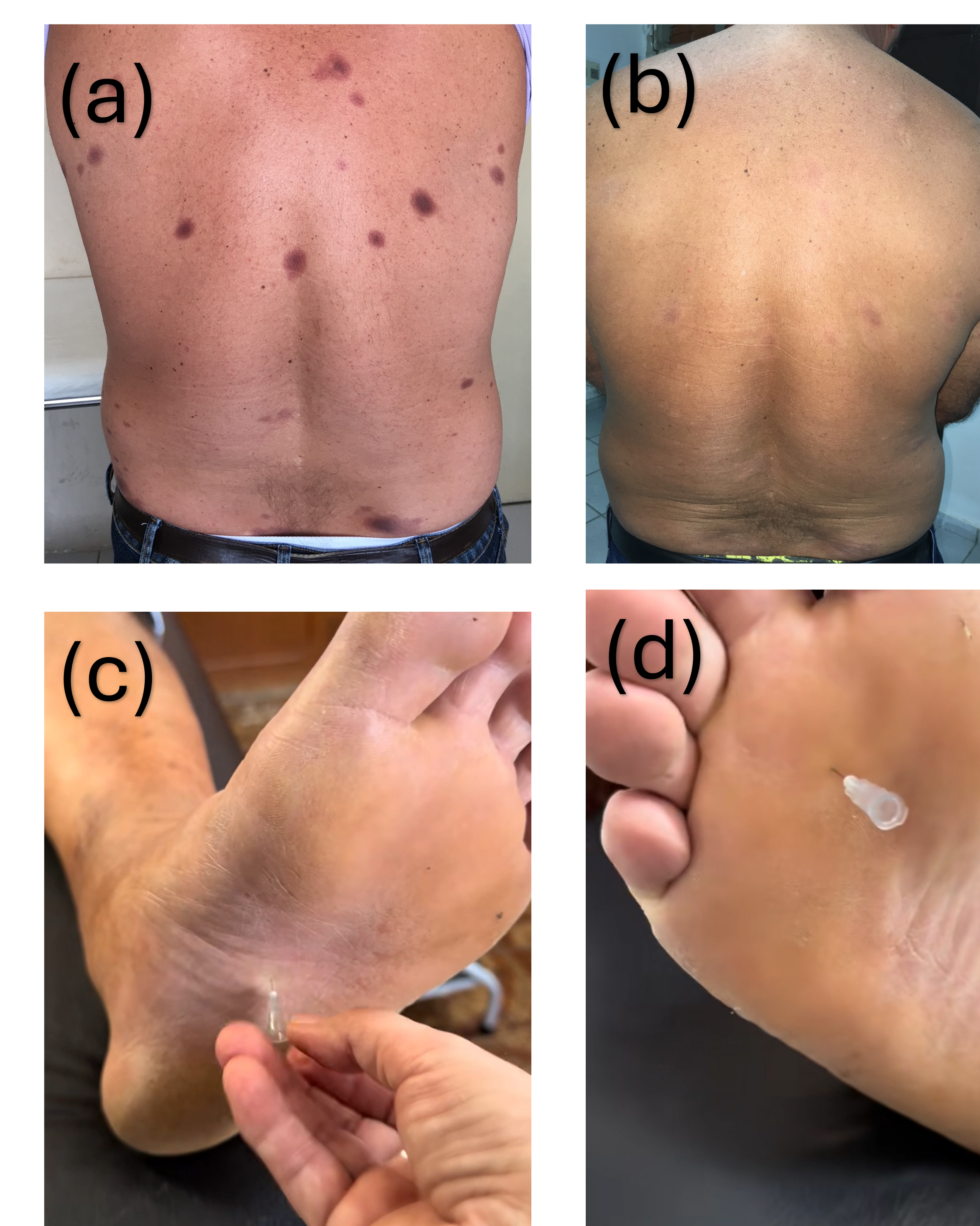

Supplement: S2 File — (TIF) [file pntd.0013616.s004.tif]
