# Supplementary material for: Therapeutic failure of multidrug therapy for leprosy: A retrospective case series in a hyperendemic Brazilian City
Source: PLoS Negl Trop Dis. 2025 Nov 25;19(11):e0013616. doi: 10.1371/journal.pntd.0013616 (PMC12646476; doi:10.1371/journal.pntd.0013616)
Supplement: Attachment — Submitted filename: Rebuttal Letter Andrea Maia.pdf [file pntd.0013616.s005.pdf]

Rebuttal Letter

Manuscript: Therapeutic Failure of Multidrug Therapy for Leprosy in a Hyperendemic Brazilian City
Authors: Fonseca et al.

Dear Drs. Xue, Cheng, Kamhawi, Brindley, and Reviewers,

We are grateful for your thorough, constructive feedback. In this revised manuscript, we have:

1. **Fully revised the Introduction and Discussion** to sharpen our study rationale, emphasize the gap in data on long-term neural complications after MDT, and situate our findings within WHO guidelines and recent literature.
2. **Expanded and clarified the Methods** to specify the retrospective, case-series design (January 2016–November 2023), inclusion criteria, and sampling protocols—including biopsies of normal skin over thickened nerves—plus detailed procedures for histopathology, qPCR, drug-resistance testing, and nude-mouse inoculation.
3. **We have added a "Variables" subsection** listing all clinical, laboratory, histological, molecular, and in vivo endpoints.
4. **Enhanced the Statistical Analysis** section to note that we used JASP v0.17.1 for frequency distributions only (no inferential tests; α not applicable).
5. **Representative histopathology images were included** in the results to illustrate key bacillary persistence and regression features.
6. **A representative image of clinical and bacteriological evolution after 24 MDT doses** was added.
7. **Renumbered and reformatted all references** to conform to PLOS NTD style and the order of in-text citation.
8. **Revised all Tables and Results text** to ensure category totals sum to 100 %, to explain sample losses in footnotes, and to maintain consistent terminology.
9. **Included new quantitative summaries** of continuous variables (age, biopsy interval, BI, and DPD scores) in the Results.

Below, we address each reviewer's comments point by point. Line numbers refer to the tracked-changes manuscript.

Reviewer #1

1. **Introduction, line 27:** *"Suggest not restricting disability to untreated cases."* **Response:** Revised to "Leprosy cases diagnosed late or left untreated can progress to disabilities and permanent neurological sequelae," removing any implication that disability occurs only in untreated cases (line 27).
2. **Methods, lines 163–166:** *"Define neurological complaints. Was Simplified Neurological Assessment (SNA) at diagnosis compared to post-24 doses?"* **Response:** Added under "Study population and inclusion criteria" (lines 141–163) that SNA scores at diagnosis were directly compared to those obtained

within six months after completing 24 MDT doses to define new or worsened neurological symptoms.

3. **Worsening of neural damage:** *“Was it defined solely by increase in disability grade? Suggest eye-hand-foot score and nerve counts.”*

   Response: Clarified (lines 154–156) that neural worsening was defined by new or exacerbated clinical complaints plus SNA changes, not by disability grade alone; noted the absence of eye-hand-foot scoring and serial nerve counts as a limitation.

4. **Persistent leprosy reactions:** *“Define criteria.”*
   Response: Under inclusion criteria (lines 157–163), defined “persistent reactions” as type 1, type 2, or combined episodes with neuritis that recurred or failed to resolve despite standard reaction therapy during and up to six months after 24 MDT doses.

5. **Histopathology protocol:** *“Clarify whether post-24-dose biopsies are routine.”*
   Response: Confirmed this protocol is part of SEINPE’s standard management for suspected active disease and cited the Brazilian neural leprosy guidelines [23] (lines 183–186).

6. **Biopsy of normal skin:** *“Is routine for non-lesional sampling?”*
   Response: Yes. Explained (lines 186–189) that, when lesions were absent, samples were taken from clinically normal skin over thickened or tender nerves in accordance with national guidelines [23].

7. **Resistance and activity testing:** *“Part of routine?”*

   Response: In “Collection of biological material” (line 179), it is stated that molecular resistance assays and qPCR are performed routinely on biopsy material at Instituto Lauro de Souza Lima under our leprosy reference protocol.

8. **Categorical variables (line 182):** *“Which were they?”*
   Response: Corrected typo; lines 239–240 now read “The results are presented using frequency distribution,” and a new “Variables” subsection lists all categorical endpoints.

Reviewer #2

1. **Objectives and hypothesis clarity:** *“State testable hypothesis.”*
   Response: We added a clear hypothesis at the end of the Introduction (lines 124–127): “Thus, this study aims to characterize therapeutic failure by integrating multidimensional markers of M. leprae activity—clinical, histopathological, molecular, and in vivo inoculation—in multibacillary patients completing twice the standard MDT duration, and to prompt re-examination of time-based discharge criteria.”

2. **Study design appropriateness:** *“Retrospective descriptive case series vs. cross-sectional.”*
   Response: Modified Study Design to “This is a retrospective, descriptive case-series study…” (line 130) and removed “observational” and “cross-sectional” terms.

3. **Population description:** *“Clarify SEINPE, sampling, and losses.”*

   Response: SEINPE was clarified in lines 134-135: “The 'Serviço de Infectologia em Petrolina' (SEINPE), the local where this study was conducted…”; inserted “One hundred thirty-one patients with multibacillary leprosy seen and monitored at SEINPE between January 2016 and November 2023 were selected” (lines 143-144); added a sample-size paragraph (n = 131) with no formal power calculation (lines 145-146), and described technical failures and tabulated sample losses in Tables S2–S4.

4. **Variables and statistical tests:** *“Summarize variables, tests, software, alpha.”*
   Response: Under “Variables,” listed each endpoint; in “Statistical Analysis,” noted no normality or inferential tests were applied, JASP v0.17.1 was used, and only frequency distributions were generated.

5. **Ethical/regulatory compliance:** *“Animal ethics?”*

   Response: We have added CEUA approval (CEUA/ILSL003/21) and humane euthanasia details (lines 247–248).

Reviewer #3

1. **Title revision:** *“Emphasize therapeutic failure.”*

   Response: Changed title to **“Therapeutic Failure of Multidrug Therapy for Leprosy in a Hyperendemic Brazilian City”** and defined “therapeutic failure” explicitly in Methods.

Reviewer #4

1. **Study design terminology:** *“Remove ‘observational’.”*

   Response: Addressed under Reviewer #2.

2. **Biopsy protocol:** *“Include in main text or SI.”*

   Response: Detailed biopsy and sample preparation protocols are included in Methods and SI.

3. **Data sources and variable collection:** *“Specify sources, subgroup analyses.”*
   Response: Clarified clinical data from notification forms and records, laboratory data from ILSL, and absence of inferential subgroup analyses.

4. **Histopathology, qPCR, mutation, resistance methods:** *“Step-by-step protocols.”*
   Response: Each assay now has a dedicated subheading with comprehensive methodology.

5. **Animal ethics:** *“Mention ethical issues.”*

   Response: See Reviewer #2.

6. **Results consistency and totals:** *“Ensure all tables sum to 100 %, specify classification.”*
   Response: Audited and corrected all frequency totals, specified Ridley–Jopling

classification [25], replaced “Blank” with “Not available,” and aligned text with table headings.

7. **Definition of “active disease”:** *“Define regressive disease, persistent bacilli, active lesion, nonspecific infiltrates.”*

   Response: Defined in Methods under “Definition of MDT-therapeutic failure” and “Variables”.

8. **Stratify inoculation by sampling site:** *“Dermal lesion vs. normal skin.”* **Response:** Added stratified analysis in “Results”, comparing growth rates in mice from lesional *versus* non-lesional biopsies.

9. **Quantitative variable summaries:** *“Provide mean/SD, min/max.”* **Response:** Added in Results immediately before the “Clinical and Dermatological Findings” section.

10. **Align lab results with objectives:** *“Stratify lab findings by clinical persistence.”* **Response:** Subgroup frequency tables in SI were added and referenced in Results.

11. **Discussion flow and next steps:** *“Avoid fragmented sentences; propose next studies.”*
    Response: Reorganized Discussion into coherent paragraphs and explicitly proposed next steps—prospective serial qPCR and histology studies, protocol standardization, and policy recommendations (Discussion lines 364-441).

We trust that these revisions fully address all concerns and substantially strengthen our manuscript. Thank you for your invaluable insights; we look forward to the opportunity to publish in *PLOS Neglected Tropical Diseases*.

Sincerely,

Andrea Maia F. Fonseca

On behalf of all co-authors
