# Supplementary material for: Therapeutic failure of multidrug therapy for leprosy: A retrospective case series in a hyperendemic Brazilian City
Source: PLoS Negl Trop Dis. 2025 Nov 25;19(11):e0013616. doi: 10.1371/journal.pntd.0013616 (PMC12646476; doi:10.1371/journal.pntd.0013616)
Supplement: Attachment — Submitted filename: Rebutal Letter Re-resubmission.pdf [file pntd.0013616.s006.pdf]

Rebuttal Letter

Manuscript: Therapeutic Failure of Multidrug Therapy for Leprosy in a Hyperendemic Brazilian City

Authors: Fonseca et al.

Dear Drs. Xue, Cheng, Kamhawi, Brindley, and Reviewers,

We are grateful for the thoughtful and constructive feedback on our manuscript, *Therapeutic Failure of Multidrug Therapy for Leprosy: A Retrospective Case Series in a Hyperendemic Brazilian City*. Guided by your comments, we have revised the paper to improve clarity, consistency, and methodological transparency while preserving the core results. Specifically, we (i) standardized denominators and clarified our available-case approach across text, tables, and figures; (ii) corrected and harmonized numeric results (e.g., histopathology 62/123 = 50.4%; nude mice inoculation 29/45 = 64.4%; qPCR performed in 101 patients with 96/101 positives; drug-resistance sequencing in 88 patients, with 2/88 showing validated resistance mutations); (iii) added and cross-referenced a Venn diagram to depict overlap of key clinical domains (Figure 1) and ensured consistent figure/table numbering and calls in the Results (Tables 2–5; Figures 1–3) ; (iv) consolidated operational definitions and analytic denominators in Table 1 and aligned the results narrative to summarize complex tables more accessibly, as requested; and (v) standardized terminology for neurological outcomes (WHO disability grade assessed via SNA) across the manuscript, replacing former terms for consistency with journal style and reviewer guidance. We also clarified procedural details in Methods (biopsy/slit-skin smear, qPCR criteria, and animal ethics for the nude-mice assay) to close prior gaps and ensure reproducibility.

Methods

Questions:

-Are the objectives of the study clearly articulated with a clear testable hypothesis stated?

-Is the study design appropriate to address the stated objectives?

-Is the population clearly described and appropriate for the hypothesis being tested?

-Is the sample size sufficient to ensure adequate power to address the hypothesis being tested?

-Were correct statistical analysis used to support conclusions?

-Are there concerns about ethical or regulatory requirements being met?

Reviewer #1

- *The objective of the study is clearly described: to verify disease activity in patients with remission of skin lesions, but with persistent and/or recurrent neurological reactions and symptoms, after 24 doses of multidrug therapy (MDT).*
- *The study design is clear, being a retrospective case series. As the study was a series of cases, with a convenience sample, it is not possible to carry out robust statistical tests, presenting only a descriptive statistical analysis, however it meets the study's purpose. All ethical aspects were respected in accordance with Brazilian legislation.*

R – We appreciate and are grateful for your consideration.

1. Objectives & Hypothesis:

Reviewer #1

- *The objective of the study is clearly described: to verify disease activity in patients with remission of skin lesions, but with persistent and/or recurrent neurological reactions and symptoms, after 24 doses of multidrug therapy (MDT).*
- *The study design is clear, being a retrospective case series. As the study was a series of cases, with a convenience sample, it is not possible to carry out robust statistical tests, presenting only a descriptive statistical analysis, however it meets the study's purpose. All ethical aspects were respected in accordance with Brazilian legislation.*

R – We appreciate and are grateful for your consideration.

Reviewer #2:

- *The study clearly articulates its objectives—to assess subclinical M. leprae activity in patients completing 24-month MDT. A testable hypothesis is explicitly stated in the revised introduction: "…to characterize therapeutic failure… in patients completing twice the standard MDT duration…".*

R – We appreciate and are grateful for your consideration.

2. Study Design Appropriateness:

- *The retrospective case-series design is suitable given the aim to evaluate outcomes in patients who completed MDT but showed persistent symptoms.*

R – We appreciate and are grateful for your consideration.

3. Population Description:

- *Inclusion criteria are clearly described (patients with persistent dermatological or neurological signs post-MDT in Petrolina, Brazil). The population is appropriate for investigating residual infection post-treatment.*

R – We appreciate and are grateful for your consideration.

4. Sample Size & Power:

- *While the sample includes all eligible patients (n=131), no formal power calculation was done. The authors justify it based on availability, but this limits inferential conclusions.*

R - This study is a retrospective, descriptive case-series that includes **all** eligible multibacillary patients over the study period; no a priori group comparisons or hypothesis tests were planned, so a power calculation was not applicable. To address precision, we now report **95% confidence intervals** for key proportions (Results Example): active disease on histopathology 64/123 = **52.0% (95% CI 43.3–60.7)**; positive nude inoculation 28/44 = **63.6% (95% CI 48.9–76.2)**; qPCR positivity 82/91 = **90.1% (95% CI 82.3–94.7)**. We also clarify this in **the Study size and Statistical analysis, and we note in the Limitations that the** results are descriptive rather than inferential.

5. Statistical Analysis:

- *Appropriate for descriptive case-series—JASP was used, and only frequency distributions were reported without inferential testing.*

R – We appreciate and are grateful for your consideration.

6. Ethical/Regulatory Compliance:

- *Ethical approvals and waivers for informed consent are documented in compliance with Brazilian standards and animal research ethics.*

R – We appreciate and are grateful for your consideration.

Results

Questions:

-Does the analysis presented match the analysis plan?

-Are the results clearly and completely presented?

-Are the figures (Tables, Images) of sufficient quality for clarity?

1. Analysis vs Plan:

Reviewer #1:

- *The analysis is in accordance with what was proposed in the objectives and results, as well as the tables and figures are very explanatory, clear and complete.*

R – We appreciate and are grateful for your consideration.

Reviewer #2:

- *Analyses align with the methods: histopathology, molecular testing, mouse inoculation, and clinical assessments were conducted as described.*

R – We appreciate and are grateful for your consideration.

Presentation Clarity:

- *Data are thoroughly presented, including several large tables comparing pre- and post-MDT variables. Results are transparent.*

R – We appreciate and are grateful for your consideration.

Figure/Table Quality:

- *Figures and tables are clear and support the text (e.g., histopathological images and concordance tables for inoculation results). Minor improvements (e.g., harmonizing terminology across tables) may help but aren't essential.*

R - We appreciate your consideration and are grateful for it. We have also improved the tables.

Conclusions

Questions:

-Are the conclusions supported by the data presented?

Reviewer #1:

- *Yes, the conclusions are in line with the data presented and the limitations of the study are clearly described.*
  This paper is of great relevance to the scientific community that studies and

promotes assistance for leprosy, as it raises an important question about the therapeutic failure of MDT in patients with persistent reactions and worsening of neurological complaints, suggesting that only the criterion of time and remission of skin lesions or reduction of the bacillary index, cannot be used to determine the cure in these cases.

R- We appreciate your consideration and are grateful for it.

Reviewer #2:

Supported by Data:

- *Conclusions are robustly supported: over 50% had histological or in vivo evidence of M. leprae activity post-MDT, and neurological impairment worsened despite “cure”.*

R- We appreciate your consideration and are grateful for it.

Limitations Stated:

- *Selection bias and generalizability issues are acknowledged (study limited to a single center, retrospective design).*

R- We appreciate your consideration and are grateful for it.

Impact on Field:

- *The study strongly challenges WHO's time-based discharge model and argues for biomarker-based post-treatment surveillance—highly relevant for leprosy-endemic regions.*

R- We appreciate your consideration and are grateful for it.

Public Health Relevance:

- *Well-addressed. The authors advocate for revised policies and highlight the risk of transmission and disability under current WHO protocols.*

R- We appreciate your consideration and are grateful for it.

Editorial and Data Presentation Modifications?

Reviewer #1:

- *Accept*

R- We appreciate your consideration and are grateful for it.

Reviewer #2:

- *Minor Recommendations:*
- *Improve grammar in a few areas for fluency (e.g., verb tense uniformity).*

R- Done. We revised **Methods → Statistical analysis** to consistent **past tense** and clarified phrasing. Specifically, we replaced present-tense constructions (e.g., "are summarized/are presented/are reported") with past tense (e.g., "**were summarized/were presented/were provided**") and kept the description of our approach concise. We also audited the entire **Methods** section for tense consistency. In **Results**, statements referring to what the article displays (e.g., "**Table 3 presents…**", "**Figure 2 shows…**") remain in **the present tense, while numerical findings are reported in the past tense**, as per journal style. In addition, the full manuscript was reviewed for grammar and fluency to ensure readability.

- *Ensure abbreviations are fully spelled out at first use in tables (e.g., DPD).*

R – Thank you for your suggestion. We standardized terminology by specifying in **Methods** that **SNA is the assessment,** and the **outcome is the WHO disability grade (0/1/2) derived from SNA**. Throughout the manuscript (text, tables, and figures), we now refer consistently to **"WHO disability grade (0–2)"**, with a single explanatory note at first mention: "assessed via SNA." Prior uses of "DPD" were replaced accordingly.

- *The Results text could summarize complex tables more narratively for general readers.*

R- We thank the reviewer for this valuable suggestion. In the revised Results section, we have restructured the text to provide clearer narrative summaries of the key findings, while still preserving the numerical details in the tables. This adjustment improves readability and allows general readers to follow the main trends without needing to interpret the tables in detail.

- *These are minor revisions and do not impact the core findings or validity of the study.*

R- We thank the reviewer for the positive feedback and for recognizing that the requested revisions are minor and do not affect the validity of our findings.

Reviewer #4:

- *To improve the clarity of the results presented in Table 3, for the variables “dermatological injuries” and “slit skin smear and morphological aspects”, I suggest stratifying the n/% values into present and absent subcategories.*

R- We agree and are grateful for your suggestion. **Table 3** now shows **present vs. absent** subcategories for dermatological lesions, neurological symptoms, leprosy reactions, and slit-skin smear **morphological aspects**. For morphology, we report only among exams with **BI > 0** at each time point and added a footnote with the denominators.

- *In the Introduction section, include a reference to the Brazilian states when mentioning Maranhão, Pernambuco, etc.*

R- We thank the reviewer for this suggestion. In the revised Introduction, we now specify that these are Brazilian states and improved the sentence for clarity.

Summary and General Comments

Reviewer #1:

- *This manuscript is of great relevance to the neglected tropical diseases community, especially those who provide care and research on leprosy, as it warns about the need to review the criteria for therapeutic cure of leprosy, emphasizing that the criteria of time and/or remission of skin lesions alone cannot be used in patients with persistent reactions and worsening of neural function, suggesting to these patients, histopathological, molecular and in vivo nude-mouse footpad inoculation criterion. In addition, this paper also highlights the importance of simplified neurological assessment in identifying worsening neural damage and the relevance of persistent neurological complaints, often neglected by health professionals.*

R- We appreciate your consideration and are grateful for it.

Reviewer #2:

- *This manuscript is a well-executed, novel, and significant contribution to the understanding of MDT outcomes in leprosy. It demonstrates convincingly that time-based definitions of cure are insufficient. The integration of multiple diagnostic approaches—histopathology, qPCR, inoculation—makes the study comprehensive and impactful.*
- *Strengths:*

- *Strong clinical relevance in a hyperendemic context.*
- *Multimodal assessment (clinical, molecular, in vivo).*
- *Large sample for a neglected tropical disease study.*
- *Clear writing and transparency in methods and limitations.*

R- We appreciate your consideration and are grateful for it.

- *Weaknesses:*

- *Retrospective design and lack of control group limit causal inference.*

R - We agree. Our study was intentionally designed as a retrospective **census case-series** to **describe** the frequency and phenotype of **persistent disease after completion of a 24-dose MDT** treatment cycle, at a referral center, not to test causal hypotheses or compare regimens. Accordingly, we did not perform a priori power calculations or between-group comparisons. To avoid any implication of causality, we have (i) clarified the descriptive aim in **Methods — Study design**, (ii) standardized language in the **Results/Discussion** to avoid causal verbs (e.g., “effect,” “impact”) and use neutral phrasing (“frequency,” “occurrence,” “co-occurrence”), and (iii) emphasized precision by reporting **exact binomial 95% CIs** for all key proportions (histopathology, inoculation, qPCR).

Although the absence of a control group precludes causal inference, we believe that this retrospective case-series adds value by consolidating multiple, independent lines of evidence (histopathology, nude mice inoculation, and qPCR) in a real-world cohort of patients meeting stringent eligibility criteria after standard MDT. These descriptive estimates are intended to **generate hypotheses** and **inform the design** of future prospective, controlled studies needed to determine mechanisms and comparative effectiveness.

- *No statistical comparison between subgroups (e.g., resistant vs non-resistant)*

R- We agree. This work was conceived as a **descriptive, census case-series**, and no prespecified hypothesis testing or between-group comparisons were planned. Subgroup contrasts (e.g., resistant vs non-resistant) are **post hoc**, **underpowered**, and affected by **non-uniform ascertainment** (resistance testing was available only for a subset), which increases the risks of selection bias, multiple testing, and spurious inferences. To avoid overinterpretation, we intentionally **did not conduct formal hypothesis tests** between subgroups.

- *Lack of longitudinal follow-up on clinical progression post-biopsy.*

R - We agree. This study was designed as a **retrospective, cross-sectional case-series** at the time of **post-MDT re-evaluation** (index biopsy), aiming to **describe** concurrent evidence of persistent disease (histopathology, nude mice inoculation, qPCR). **Longitudinal clinical follow-up was not protocolized,** and ascertainment after the index visit was heterogeneous; therefore, we did not analyze post-biopsy progression, relapse, disability trajectories, or response to re-intervention. To avoid overstating inference, we have (i) tempered language throughout to emphasize **point-in-time characterization**, (ii) expanded the **Limitations** to explicitly state that the absence of standardized follow-up **precludes conclusions about clinical trajectory**, and (iii) directed readers to future **prospective cohort/registry designs** with scheduled assessments as the appropriate framework to quantify progression and comparative effectiveness.

Reviewer #3:

- *Therapeutic Failure of Multidrug Therapy for Leprosy in a Hyperendemic Brazilian City*
  Title: I recommend think about a new title indicate the study design Suggestion: Therapeutic Failure of Multidrug Therapy for Leprosy: A Retrospective Case Series in a Hyperendemic Brazilian City

R- We agree. We changed the title as suggested.

- *This is an extensive case series. Why did the authors not include only the cases with complete data at both diagnosis and discharge*

R- We appreciate the suggestion. Our analytic target was **point-in-time characterization at the post-MDT re-evaluation (index biopsy)**, i.e., concurrent evidence of persistent disease (histopathology, nude mice inoculation, qPCR) **after completion of 24-dose MDT**. Requiring *paired completeness at both initial diagnosis and MDT discharge* would have (i) **substantially reduced the sample**, and (ii) likely **introduced selection bias** because missingness is **not at random** in this setting: as a referral center, many patients initiated/finished MDT at external facilities, and historical records vary across years and sites. This choice increases transparency by reflecting operational challenges in real-world practice, which we consider a key strength of this case series.

3. Abstract:

- *I recommend that the authors include the data collection period in the abstract Methods section. This is important to contextualize the findings, particularly given the retrospective nature of the study.*

R- Thanks for your observation. We agreed, and we added the study period to the abstract methods section (and in the author summary) to contextualize the retrospective, census case-series design.

- *From lines 125 to 128. I Irecommend aligning the two objectives so they convey the same scope. A suggestive goal for unify them.*
- *Suggestive: We aimed to characterize therapeutic failure and assess subclinical M. leprae activity in multibacillary patients who completed an extended 24-dose MDT course, in order to re-evaluate the adequacy of time-based discharge criteria.*

R- We agree and have adjusted as suggested.

4. Methods

- *In the section study design*

- *I recommend combining the STROBE (Strengthening the Reporting of Observational Studies in Epidemiology) and CARE (CAse REport) guidelines to improve the reporting quality of your case series. While CARE enhances clinical detail and patient-centered information, STROBE provides a clear and structured framework for reporting observational studies such as this one.*

R- We agree. The Methods—Study design and reporting section now states that reporting adheres to STROBE and incorporates selected CARE elements adapted to a case-series (aggregate patient information and a cohort-level timeline). We also added STROBE (S1) and CARE (S2) checklists with item-to-section cross-references, and a cohort-level timeline in the Supporting Information. No protocol details were removed.

- *Upon reviewing the Methods section, I noticed that key elements such as study setting, population, inclusion criteria, and clinical procedures are mixed together.*
- *I recommend grouping the information regarding the study type, study site, data collection period, and location of laboratory analyses under a unified subheading titled: "Study Design and Setting"*
- *This subheading should include:*

- *The type of study (retrospective case series); The institution or location where the study was conducted; The time frame during which data were collected: The site(s) where laboratory analyses were performed*

R- We appreciate your suggestion. Done. We created a unified **“Study Design and Setting”** subsection specifying the study type (retrospective census case-series), clinical site(s), data-collection period, and laboratory locations. We also separated **Participants and eligibility** and **Data sources and measurement** to prevent mixing of inclusion criteria and clinical procedures with design elements. No protocol details were removed.

- *From lines 137 to 140 I recommend relocating this sentence to other subheading*

R- We agree and have adjusted as suggested. To avoid mixing study design with clinical procedures, we moved the procedural description from **Study Design and Setting** to **Methods → Data sources and measurement**, under the new subheading **“Clinical sampling at the post-MDT index re-evaluation.”** This aligns with STROBE by separating design/site information from measurement procedures. No protocol details were removed.

- *Lines 145 to 146: This data is not recommended in this section since refers o the period of data collection*
- *“No formal sample-size calculation was performed, and the sample reflects the complete available cohort”.*
- *This sentence is not needed since the case series do not allow inferences*

R- Agreed. We **removed** the sentence “No formal sample-size calculation was performed, and the sample reflects the complete available cohort.” To avoid mixing content, information about the **data-collection period** is reported only under **Methods — Study Design and Setting**. The **Participants and eligibility** subsection already states that **all eligible multibacillary patients (n=131)** were included during the study period, so a separate study-size statement is unnecessary for this descriptive case series.

- *I suggest rewriting the inclusion criteria in the form of a continuous paragraph, with each criterion separated by semicolons rather than bullet points Recommendation: inclusion criteria required the presence of at least one of the following findings: (1) erythematous or infiltrated skin lesions observed after completion of MDT, in comparison to the clinical presentation at diagnosis; (2) persistence, worsening, or new onset of neurological symptoms—such as muscle cramps, paresthesias, or numbness in the extremities (hands and feet)—*

accompanied by acute or chronic pain, burning sensations, or spontaneous sensory disturbances; (3) deterioration of neural function documented by the Simplified Neurological Assessment (SNA), compared to findings at diagnosis; (4) persistent leprosy reactions, including type 1 or type 2 reaction episodes, or both, with or without neuritis, occurring separately or simultaneously, unresponsive to conventional treatment during or after MDT; these reactional episodes were documented either at discharge or within six months following treatment completion and coincided with the time of skin biopsy.

R- Thank you. We restructured **Methods** to avoid mixing design/setting with eligibility. The **setting** is now reported under **Study Design and Setting** (type of study, clinical site(s), laboratory locations, and period). To present the cohort and entry criteria clearly—and to align with **STROBE item 6 (Participants)**—we use the subheading **"Participants and eligibility"** rather than "Setting and participants."

We also implemented your formatting request: the **inclusion criteria are written as a continuous paragraph with semicolons** separating each criterion. If the editor prefers the label **"Inclusion criteria"**, we are happy to adopt that wording; the content will remain identical.

- *Variables*
- *Please describe each variable and specify the corresponding content under the appropriate subheadings in the Methods section. Additionally, present the different proportions or distributions for each variable in the Results section*

R- Done. Each variable is now defined under its corresponding **Methods** subheading with operational definition, measurement/source, and analytic denominator (see *Clinical sampling/dermatological examination; Neurological assessment (SNA); Slit-skin smear—BI; Slit-skin smear—morphological aspect; Histopathology; Nude mice inoculation; PCR analysis (qPCR);* and ***M. leprae* sequencing***). Operational definitions and denominators are **consolidated in Table 1**. The corresponding **Results** report the distributions for each variable (**Table 3** and **Figure 2**), histopathology–inoculation **concordance in Table 4**, and locus-level genotyping in the **Supporting Information**.

- *Definition of MDT-therapeutic failure*
- *From lines 176 to 179 authors stated "MDT-therapeutic failure was defined as any clinical (erythematous/infiltrated lesions, persistent reactions, neurological complaints, or worsening on simplified neurological exam) or histopathological (presence of viable M. leprae in skin or nerve biopsy) evidence of active disease*

after 24 MDT doses. Specify the reference for definition of MDT therapeutic failure (it would be this - Brasil. Ministério da Saúde. Protocolo Clínico e Diretrizes Terapêuticas da Hanseníase. Brasília: MS; 2022 or another?)

R- We now cite the Brazilian Ministry of Health Technical-Operational Manual (2016), which is the only national guideline that provides explicit operational criteria for MDT-therapeutic failure. In this document, failure is suspected when there is no clinical improvement during adequately indicated MDT (6 or 12 doses) and, in multibacillary patients who have completed 24 doses, when there is clinical activity at discharge and/or the presence of intact bacilli on slit-skin smear or skin histopathology. The more recent Clinical Protocol and Therapeutic Guidelines (PCDT 2022) does not explicitly define therapeutic failure, remaining silent on this point, but it does not contradict the 2016 guidance. Therefore, our operational definition follows the Technical-Operational Manual (2016), which remains the most straightforward and most practical reference for clinical and programmatic use.

We intend to report the reality seen in clinical practice: a substantial fraction of multibacillary patients show ongoing disease at the end of the standard 24-dose MDT course, documented by histopathology, nude mice inoculation, and qPCR. We therefore use the term therapeutic failure deliberately and transparently, consistent with previous Brazilian national guidance for post-MDT assessment, to avoid mislabeling such cases as “cure” or “relapse” and to center patient outcomes.

While MDT has historically enabled programmatic scale-up, our data indicates that its current implementation is insufficient for a meaningful subset of patients. We believe that recognizing and naming therapeutic failure is essential to reducing avoidable morbidity and to motivating prospective studies and policy revision of discharge criteria and retreatment pathways.

- *Statistics analyisis*
- *From lines 239 to 242, the authors describeb data handling and analysis using a mix of tenses and slightly unclear phrasing. I recommend the use of the past tense, as it describes actions that were already completed.*

R- Done. We revised the **Methods—Statistical analysis** text to use consistent **past tense** and clarified phrasing. Tracked changes are visible in that subsection.

- *From lines 239 to 242, the authors describeb data handling and analysis using a mix of tenses and slightly unclear phrasing. I recommend the use of the past*

tense, as it describes actions that were already completed. For instance rewrite - are presented (line 241) as were presented.

R- Done. We revised **Methods → Statistical analysis** to consistent **past tense** and clarified phrasing. Specifically, we replaced present-tense constructions (e.g., "are summarized/are presented/are reported") with past tense (e.g., "**were summarized/were presented/were provided**") and kept the description of our approach concise. We also audited the entire **Methods** section for tense consistency. In **Results**, statements referring to what the article displays (e.g., "**Table 3 presents…**", "**Figure 2 shows…**") remain in **present tense**, while numerical findings are reported in **past tense**, as per journal style.

- *Ethical*
- *From line 244, the phrase "as required by" should be replaced with "established by" to avoid redundancy and include the study type for instance this case series.*

R – Done. We revised "Ethical Considerations". The requirements were implemented.

- *From lines 248 to 249, clarify the reason why the Ethical Committee on Experimental Animals was involved. Was it because the human tissues were injected intradermally into the hind footpads of athymic mice? What was the reason? Goal?*

R - Yes. The animal ethics review (CEUA/ILSL 003/21) was required because the study included an **in vivo nude mice footpad inoculation assay** using suspensions prepared from human biopsy tissue obtained at the post-MDT index visit. The **goal** was to assess the **viability of *M. leprae*** at discharge, as the organism cannot be cultured in standard media. **At the predefined endpoint, mice were humanely euthanized to permit terminal harvest of the inoculated footpads for bacillary recovery and viability assessment**, with anesthesia, minimization of pain/distress, and humane endpoints per the CEUA-approved procedures. We added clarifying sentences in **Methods—Nude mice inoculation** and **Ethical**

- *Important: some parts of methods were written in presente tens, however, The methods sections should be written in the past tense, since they describe actions that had occurred. See:*
- *Lines 191 to 193: "The nerves are selected by identifying...."*
- *Lines 240 to 241: "The results are presented as frequency distributions"*

R- We agree. We revised the **Methods** to consistent **past tense** (e.g., “are presented” → “were presented”) and audited the section for tense consistency. In **Results**, present tense was retained only when referring to what the paper displays (e.g., “Table 3 presents…”), per journal style.

- *I recommend that the table 2, currently placed in the Methods section, be moved to the Results section and presented immediately after the first paragraph*

R- We appreciate the suggestion. This table **summarizes operational definitions and analytic denominators**, not outcomes; per **STROBE** and to avoid mixing design/measurement with findings, we **retained it in Methods**. To improve navigation, the **first Results paragraph now explicitly points to this table**, and all distributions/proportions are presented in **Results** (**Table 4** and **Figure 2**), with histopathology–inoculation concordance in **Table 5**. We also revised Table 1 to improve clarity. We note that another reviewer requested the opposite (placing it in Methods). If the editor prefers relocation, we are happy to move the table to the start of Results with unchanged content.

- *I recommend reducing redundancy between the text (lines 260–264) and Table 1 by removing the detailed counts and percentages for sex and age groups from the narrative. Instead, highlight only key findings (majority male, peak age 40–59 years). Sentences such as - Detailed socio-demographic and age distribution data are shown in Table 1 – could help authors reducing the redundancy. Keep your eyes in the number of tables, since it will change after corrections. The same recommendation for clinical forms and leprosy reactions descriptions in table1.*

R- We appreciate the suggestion. We condensed the narrative to highlight key points (majority male; age peak 40–59 years) and retained detailed counts/percentages in **Table 2**. We applied the same approach to clinical forms and reactional episodes, now in **Table 3.** (n. Cross-references and table numbering were updated accordingly).

- *From lines 281 ot 292 I recommend adding a Venn diagram to illustrate the overlap between the different clinical presentations described (persistent skin lesions, neurological deterioration, and reactional episodes with neuritis). This would help readers visualize how many patients experienced more than one clinical feature simultaneously, rather than interpreting each finding in isolation.*

R- We thank the reviewer for this helpful suggestion. We have now generated a Venn diagram, incorporated as **Figure 1**, to illustrate the overlap between persistent skin

lesions, neurological deterioration, and reactional episodes with neuritis (page 16). Neurological deterioration was defined as either worsening **WHO disability grade measurements** or the presence of neurological symptoms at discharge, to ensure consistency with terminology used throughout the manuscript. The figure highlights how these clinical features co-occurred in individual patients, complementing the frequencies reported in the text. We also revised the Results section to explicitly reference this figure (page 14) and provide detailed counts in Table 4.

- *From lines 294 to 321, I recommend clarifying whether the 14 patients who did not undergo slit-skin smear at diagnosis are the same individuals who also lacked histopathology or if they are different participants. If these are patients without either of these essential diagnostic tests, it might be more appropriate to exclude them from the analysis, as missing such critical baseline data could compromise the accuracy of the parameters being described. Given that this is a relatively large case series (n = 131), excluding patients with incomplete diagnostic data would likely not affect the overall descriptive analysis and would strengthen the reliability of the findings. Alternatively, the authors could explicitly justify the inclusion of these patients despite the missing data.*

R- We thank the reviewer for raising this critical point. We have clarified in the *Histopathology* subsection (page X, lines Y–Z) that the 14 patients without slit-skin smear at diagnosis were **not the same individuals** whose histopathology could not be assessed due to technical failure (8 specimens). In addition, a few patients also lacked slit-skin smear at the time of discharge, which is reported separately in the follow-up results.

Importantly, our case series was designed to include **all 131 consecutive patients** who completed 24 doses of MDT during the study period. We considered that excluding patients with incomplete baseline tests would not only reduce sample size but also introduce selection bias, underestimating the frequency of technical limitations encountered in real-world practice. Clinical information from these patients remained valid and contributed to the descriptive analysis of post-treatment outcomes.

Therefore, all patients were retained in the analysis, and the missing data are explicitly acknowledged as limitations. This approach aligns with the descriptive design of the study and enhances the external validity of the findings.

- *I recommend revising the sentence from lines 323to 330, as it is too long and should be divided into at least three shorter sentences to improve it. Additionally, the statement from lines lines 329–330 as follow "These results indicate that*

known M. leprae mutations were not the primary drivers of therapeutic failure in the MDT-treated cases presented in this study" does not present a result but rather an interpretation. This portion would be more appropriately placed in the Discussion section.

R- We thank the reviewer for this observation. We have revised the *Results* section to present the data in shorter sentences and limited this section to objective findings only. The interpretative statement regarding the low frequency of resistance-associated mutations has been moved to the *Discussion*. This adjustment clarifies the distinction between descriptive results and interpretive discussion, as recommended.

- *Observing the table 3, it was better if the same sample size (n) was the same for both time points assessed, diagnostic and after MDT, to allow a direct comparison and to clearly determine whether the bacteriological index, disability grade increased, and proportion of intact bacilli keep the same the end of treatment. Even though the sample may be decreased, I recommend excluding patients with incomplete data. Given the large total sample size (n = 131) it will not affect the research.*

R- [Table 3 = Table 4 in current version]. We thank the reviewer for this thoughtful suggestion. While we agree that equal denominators at both timepoints would facilitate direct comparisons, we intentionally chose not to exclude patients with incomplete data. This study was designed as a **descriptive case series of all 131 consecutive patients** who completed 24 doses of MDT. Missing data were mainly due to technical limitations or patient refusal, which are relevant operational challenges in real-world practice. Excluding these patients would reduce representativeness and introduce selection bias.

To address this, we ensured **full transparency of analytic denominators**: Table 4 now explicitly reports the sample size (n/N) available for each parameter at each timepoint, and footnotes clarify that denominators vary due to missing data. This allows readers to interpret the results accurately without artificially reducing the cohort.

Thus, all 131 patients were retained in the analysis, denominators are shown for each parameter, and the limitations associated with missing data are acknowledged in the Results and Limitations sections.

- *The authors made the changes recommended by the reviewer. There were significant improvements in the clarity of the manuscript, especially in the Methods and Results sections.*

R- We sincerely thank the reviewer for the positive feedback and are pleased that the revisions improved the clarity of the *Methods* and *Results* sections. We appreciate the constructive comments that guided these improvements.
